# The Institutional Library

**Published:** 1914-10-03

**Authors:** 


### THE INSTITUTIONAL LIBRARY.

### The Literature of First Aid.

The effect of the war on the publishing trade in general has been, to common knowledge, sufficiently disastrous. But to the results of the blowing of any ill-wind there are always exceptions, and it is very obvious that the publishers of First Aid and Red Cross manuals have had nothing to complain of. It is, indeed, no exaggeration to say that a very considerable harvest has been reaped already in this field. Fortunately for the students of these works the majority of the text-books in vogue are revised editions of pre-existing manuals of established merit and containing authoritative information. Were this not the case, and were the market to be flooded with a mushroom growth of ill-digested and probably antiquated matter on the subject of the treatment of the wounded, the harm that might be done by the enthusiastic ladies who flock to the classes now being held everywhere would possibly be great. There seems, however, but little likelihood of amateurs being employed in any work more serious than that of attending to convalescents. The First Aid and Red Cross manuals which are most popular at present are works of considerable merit both from the military and from the surgical point of view. The necessity for uniformity is perhaps not conducive to keeping pace with the progress of surgical science in the matter of details, for regulations cannot be altered too frequently. Hence we find that certain well-worn methods are still retained, although neater and less cumbersome ones are available. To take one instance. The method of sterilising the skin by the simple application of tincture of iodine is not sufficiently emphasised in most of the works we have seen, and the excellent results of this fluid when applied to any wound, even directly after infliction on the battle-field, are not mentioned. This is merely one example of the difficulty of keeping up to date official handbooks, whose regulations are designed for those with no previous medical knowledge.

### Red Cross Training Manuals.

Manual for Women's Voluntary Aid Detachments. By P. C. Gabbett, M.R.C.S., Lieut.-Col. I.M.S. (Ret.). (Bristol: John Wright & Sons, Ltd. London: Simpkin, Marshall and Co., Ltd. 1913. Second Edition, Revised and Enlarged. Reprinted 1914. Price 1s. net.)

Members of women's Voluntary Aid Detachments have already passed examinations in "first aid" and in "home nursing," but they are not proficient to undertake the specialised nursing that is necessary in a temporary hospital for the care of sick and wounded soldiers. The excellent instruction given in Red Cross training manuals regarding improvisation and field-work does not, after all, provide for the chief part of women's duties, which lie in the hospitals, and not in the field and transport work—this may be left to the men's section. The little manual now under notice will be found full of practical matter bearing on the true object of the existence of women's detachments—namely the care of the sick and wounded in time of war. A particularly useful chapter is that on the equipment of temporary hospitals, in which a detailed list is given of all the articles required for the equipment of a thirty-bed hospital, including furniture, kitchen outfit, and surgical appliances. It is surprising what a wide range of information is covered by the contents of this book—there are notes on nursing, on first aid, on operations and asepsis, on diets, and on training in camp. It is obvious that this book is one of the most useful of the "war manuals," and distinctly of the greatest value to women who desire to become really efficient helpers.

### The History of Bethlem Hospital.

The Story of Bethlehem Hospital from its Foundation in 1247. By Edward Geoffrey O'Donoghue, Chaplain to the Hospital. (London: T. Fisher Unwin, 15s. net.)

We turned to this volume with pleasant anticipation knowing that there has probably been no one better equipped with love for, and knowledge of, the past of "Bedlam" than Mr. O'Donoghue, who has made research into its origins and development a part of his life's work. In this respect he has set an example to institutional chaplains, besides doing a service to the hospital world. When Simon Fitz Mary, the neglected founder, in 1247 made his deed of gift of the land where Liverpool Street Station now stands, he was not thinking of a hospital for the care of disease, but rather of a priory where masses should be sung for the repose of his soul, and thus the story of the opening centuries of Bethlem Hospital has little to do with hospitals in our sense of the word. The life of the institution for the first hundred years is shrouded in doubt, but Mr. O'Donoghue disinters evidence of poverty and Royal licences for the brothers to beg for their needs, till in 1346 the protection of the city was accorded by formal agreement to the unfortunate institution. In later times Bethlem became less anxious to invoke the aid, with its accompanying rights of representation or inquisition, of public authorities. But gradually the purely religious work of the hospital became overshadowed by its care for the sick, and John Arundell may be described as its first medical superintendent, in 1457. Indeed, before 1403 the hospital possessed a house at Charing Cross, and the author presumes that "it was the asylum from which certain insane patients were transferred to Bethlehem Hospital as its first insane patients." This would date the beginning of its mental work from 1377. Treatment at this time included ducking and "the use of ligatures and whips," apart from the ministrations of the mediæval exorcist. But unworthy officers still impeded the hospital's progress, and an amusing story is told of the many rascalities of Peter Taverner, janitor and treasurer between 1388 and 1403. Stocks, manacles, and iron chains are significantly mentioned as among the many belongings of the hospital which he was accused of stealing, while he feathered his nest by appropriating legacies and charging the patients and their friends for the barest necessities. He was deprived of his office, but apparently otherwise made good his escape. Leaving mediæval times, an important date is 1547, when the City bought back the "custody and patronage of the hospital." It will be remembered that monasteries and hospitals about this time had been put into straits by the Royal policy. With the sixteenth century begins the era of the hospital as one of the sights of London, and it soon became a centre to which all and sundry resorted.

Luckily some of these were the choice and master spirits of their age, like Hogarth, to whom, when we come to the eighteenth century, a chapter is devoted. But, confining ourselves to the hospital side of the story, we recommend readers to turn to the chapters on Dr. Crooke, whose neglect led to two Commissions under Charles I., and also to the appointment of stewards in the place of masters. The later chapter on Dr. Tyson is still more interesting. In 1662 a matron for the female patients was appointed, and a special ward set apart for their use, but the early matrons were little more successful than the early masters. Pepys, by the way, was a governor at this time. The hospital escaped the Great Fire, but in 1674 the governors decided to move, and Robert Hooke was appointed architect of the second hospital in Moorfields. Dr. Tyson was succeeded by Dr. Richard Hale, who considered company advantageous to patients, and was apparently the first to believe in the value of music, which plays an important part in Bethlem to-day. In 1752 a picture of the life at Bethlem is given in that curious volume, 'Low Life : or One Half the World Knows Not how the Other Half Live," from which extracts appear in the chapter called "The Betsy Prig School of Nursing." Faults in the structure of the building led to the erection of the third Bethlem Hospital in St. George's Fields, Southwark, where the foundation-stone was laid in 1812. Three years later 120 patients were brought to their new quarters. The last chapters of the book describe in detail the present hospital, and sketch the revolution in the treatment of mental disease which far-sighted reformers like Tuke, Pinel, and others forced on the "asylums" during the nineteenth century. This section will perhaps prove the most interesting to modern institutional readers, but we have preferred, in the limits of a brief review, to touch rather on some points of Bethlem's long history, which makes it unique among our institutions. The volume, which has clearly been a labour of love, differs therefore in this respect from most hospital histories : it is not confined to the evolution of a hospital as such, but touches the history of England at a hundred points, and has associations with famous men of every type for several centuries. This has given the author his opportunity to do more than record the facts of past annual reports or courts of governors, and he has taken full advantage of it.

The Child's Diet. By J. S. Curgenven, M.R.C.S., M.R.C.P. (London : H. K. Lewis. Second Edition. Pp. 115. Price 2s. 6d. net.)

We have looked carefully through this book, of which the first edition was issued several years ago, and can recommend it as an elementary guide to parents and nurses in the subject of which it treats. The information given is trustworthy—though there are one or two minor points over which the bulk of expert opinion is against the author—and conveyed in a form which any intelligent layman can be expected to assimilate without misunderstanding. Above all, it is practical.

